# RNA-Binding Proteins Implicated in Mitochondrial Damage and Mitophagy

**DOI:** 10.3389/fcell.2020.00372

**Published:** 2020-06-04

**Authors:** Stylianos Ravanidis, Epaminondas Doxakis

**Affiliations:** Center of Basic Research, Biomedical Research Foundation, Academy of Athens, Athens, Greece

**Keywords:** mitochondria, RNA-binding proteins, TDP43, FUS, TIA1, TIAR, PUM, mitophagy

## Abstract

The mitochondrial lifecycle comprises biogenesis, fusion and cristae remodeling, fission, and breakdown by the autophagosome. This cycle is essential for maintaining proper cellular function, and inhibition of any of these processes results in deterioration of bioenergetics and swift induction of apoptosis, particularly in energy-craving cells such as myocytes and neurons. Regulation of gene expression is a fundamental step in maintaining mitochondrial plasticity, mediated by (1) transcription factors that control the expression of mitochondrial mRNAs and (2) RNA-binding proteins (RBPs) that regulate mRNA splicing, stability, targeting to mitochondria, and translation. More recently, RBPs have been also shown to interact with proteins modulating the mitochondrial lifecycle. Importantly, misexpression or mutations in RBPs give rise to mitochondrial dysfunctions, and there is strong evidence to support that these mitochondrial impairments occur early in disease development, constituting leading causes of pathogenesis. This review presents key aspects of the molecular network of the disease-relevant RBPs, including transactive response DNA-binding protein 43 (TDP43), fused in sarcoma (FUS), T-cell intracellular antigen 1 (TIA1), TIA-related protein (TIAR), and pumilio (PUM) that drive mitochondrial dysfunction in the nervous system.

## Introduction

Adenosine triphosphate (ATP) production by mitochondria is essential for most cellular activities. In addition to ATP generation, however, mitochondria are heavily involved in calcium homeostasis, production and modulation of reactive oxygen species (ROS), and in the execution of apoptosis.

Mitochondria are highly dynamic organelles characterized by rapid movement and undergo some five fusion-fission cycles every hour to properly maintain their function (Twig et al., 2008; Pernas and Scorrano, 2016). Mitochondrial fusion is the process in which mitochondria fuse together to spread metabolites, proteins, and DNA throughout the network to maintain mitochondrial (mt) DNA replication and oxidative phosphorylation (OXPHOS) capacity (Chen et al., 2005, 2010; Silva Ramos et al., 2019). It is mediated by optic atrophy 1 (OPA1), and mitofusin-1 and 2 (MFN1/2) (Chen et al., 2003; Olichon et al., 2003). Mitochondrial fission, on the other hand, is the process in which mitochondria divide to separate dysfunctional/depolarized mitochondrial sections in a daughter mitochondrion that will be targeted by autophagy, otherwise known as mitophagy (Twig et al., 2008). It is primarily regulated by dynamin-related protein 1 (DRP1) and dynamin-2 (DYN2) with the aid of adaptor proteins mitochondrial fission 1 (FIS1), mitochondrial fission factor (MFF), and mitochondrial dynamics proteins 49 and 51 (MiD49/51) (Smirnova et al., 1998; Yoon et al., 2003; Gandre-Babbe and van der Bliek, 2008; Otera et al., 2010; Palmer et al., 2011; Lee et al., 2016). Additionally, folds of the inner membrane of the mitochondrion (known as cristae) that are formed to increase the surface area for housing the electron transport chain (ETC) complexes and ATP synthase continuously remodel to improve mitochondrial function (Enriquez, 2016). Collectively, these mitochondrial morphology events comprise the mitochondrial life cycle.

Mitochondrial dynamics are altered according to the energy requirements of the cell, nutrient availability, stress, and aging, and depend on transcriptional and post-transcriptional mechanisms. While transcription factors mediate the expression of nuclear and mitochondrial genes, RNA-binding proteins (RBPs) regulate splicing, stability, localization, and translation events. More recently, RBPs have been shown to interact directly with proteins on mitochondrial surface, too. In this review, we present findings that implicate RBPs misregulation in mitochondrial damage. We focus on transactive response DNA-binding protein 43 (TDP43), fused in sarcoma (FUS), T-cell intracellular antigen 1 (TIA1), TIA-related protein (TIAR), and pumilio (PUM), as there is substantial experimental data that show their involvement in mitochondrial pathology. General features, such as the neurological symptoms associated with their perturbation, molecular and cellular function, target mRNAs and subcellular localization have been described in our previous review, and are thus not described here (Ravanidis et al., 2018).

## TDP43

**FIGURE 1:**
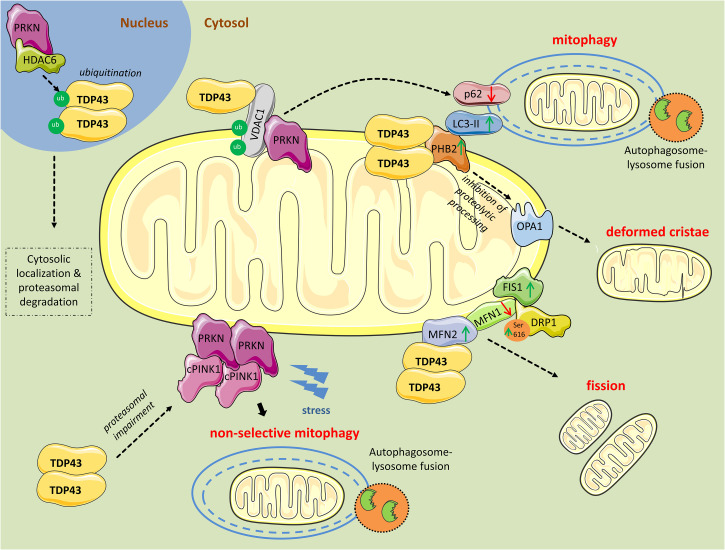
Mitochondrial perturbations induced by TDP43. PRKN in complex with HDAC6, ubiquitinates nuclear TDP43 promoting its cytoplasmic localization and proteasomal degradation. However, as revealed from research in aging or neurodegenerative diseases, TDP43 often persists in the cytosol and forms aggregates. Excess cytosolic TDP43 interacts with VDAC1, located in the outer mitochondrial membrane, but it is still unclear if interferes with its functions. Polyubiquitination of VDAC1 by PRKN is essential for driving mitophagy. Moreover, cytosolic TDP43, translocated to the outer mitochondrial membrane, directly interacts with PHB2 and, in parallel, increases its protein levels. PHB2 is known to interact with LC3-II to induce mitophagy. PHB2 is also involved in mitochondrial membranes fusion by stabilizing indirectly the long forms of OPA1. Additionally, TDP43 directly interacts with MFN2, a mitochondrial membrane protein regulating mitochondrial fusion, and possibly stabilizes its expression. Concurrently, TDP43 leads to reduced levels of another fusion protein, MFN1, and increases levels of FIS1 and DRP1 phosphorylated at Ser616, proteins promoting mitochondrial fission. Finally, TDP43 downregulates *PRKN* mRNA and protein levels, and impairs the proteasome, leading to the accumulation of cleaved PINK1 (cPINK1) in the cytosol. During stress conditions cPINK1 aggregates recruit PRKN to the mitochondria launching mitophagy in otherwise healthy mitochondria (non-selective mitophagy).

Mutations or deregulation of transactive response DNA binding protein 43 (*TDP43* or *TARDBP*) expression have been associated with a spectrum of neurodegenerative diseases including frontotemporal lobar degeneration (FTLD) and amyotrophic lateral sclerosis (ALS) (Ravanidis et al., 2018). Electron microscopy (EM) analyses of patient brain samples as well as cellular and animal models of TDP43 proteinopathy revealed prominent mitochondrial impairment, including abnormal cristae architecture and diminished cristae surface area (Wang et al., 2019). Further, increased TDP43 expression induced mitochondrial dysfunction, including decreased mitochondrial membrane potential and elevated production of ROS (Wang et al., 2019; Figure 1).

Alzheimer’s disease (AD) pathology includes mitochondrial perturbations such as alterations in respiratory function, mitochondrial biogenesis, and mitophagy (Cai and Tammineni, 2017; Chakravorty et al., 2019). Using the APP/PS1 transgenic mouse model co-expressing the familial AD Swedish mutations (APP^*K*595*N,M*596*L*^) and mutant human presenilin 1 (PSEN1-ΔE9) under stress conditions, Davis et al. (2018), found increased accumulation of the N-terminal (27 kDa, N27) and C-terminal (30 kDa, C30) fragments of TDP43 in mitochondria. Immunoprecipitation from cortex lysates, to reveal the interacting partners of TDP43, showed enrichment for mitochondrial proteins, including prohibitin-2 (PHB2) and voltage-dependent anion channel 1 (VDAC1). PHB2 is a scaffold protein and a mitophagy receptor located in the inner mitochondrial membrane. It is involved in targeting mitochondria for autophagic degradation by interacting with microtubule-associated protein 1A/1B-light chain 3 (LC3) conjugated to phosphatidylethanolamine (LC3-II), which is found in autophagosomal membranes (Lahiri and Klionsky, 2017; Wei et al., 2017). Accordingly, PHB2 knockdown was shown to drastically reduce mitochondrial clearance (Wei et al., 2017). In addition, PHB2 is involved in mitochondrial membranes’ fusion by stabilizing indirectly the long forms of dynamin-like GTPase OPA1, which mediates mitochondrial inner membrane fusion and cristae morphogenesis. Loss of PHB2 impairs the stability of OPA1, affects mitochondrial ultrastructure, and induces the perinuclear clustering of mitochondria (Merkwirth et al., 2012). Overexpression of TDP43 was found to increase PHB2 levels, whereas TDP43 knockdown reduced PHB2 and LC3-II expression in HEK293T cells treated with carbonyl cyanide m-chlorophenylhydrazone (CCCP), an inducer of mitophagy (Davis et al., 2018). Accordingly, an increase in the E3 ubiquitin ligase parkin (PRKN)-positive punctate staining (indicative of mitophagy) in cells treated with CCCP was observed, which was enhanced with TDP43 overexpression and reduced when TDP43 levels were knocked down (Davis et al., 2018). In parallel with these findings, in NSC34 cells that exhibit motor neuron features, overexpression of full length or C-terminal fragments of TDP43 (TDP25 and TDP35) led to increased levels of LC3-II and decreased levels of autophagy receptor p62 (SQSTM1) (Hong et al., 2012). Collectively, these results suggest that TDP43 overexpression is linked to enhanced mitophagic flux.

TDP43 expression also affects mitochondrial dynamics. Using transgenic mice expressing full-length human TDP43, Xu et al. (2010) observed aggregates of mitochondria, with decreased cristae and vacuoles within the mitochondrial matrix, adjacent to the nucleus, accompanied by enhanced levels of FIS1 and pro-fission phosphorylation of DRP1 at Ser616, both key mediators of the mitochondrial fission machinery (Taguchi et al., 2007). Conversely, a marked reduction in MFN1 expression, which plays an essential role in mitochondrial fusion, was observed (Xu et al., 2010).

Corroborating evidence came from Wang et al. (2013), showing that overexpression of wild-type TDP43 in primary motor neurons reduced mitochondrial length and density in neurites. Further, transgenic mice overexpressing wild-type or mutant TDP43 displayed significantly shorter, smaller, and damaged mitochondria (Wang et al., 2013). In contrast, artificial miRNA-mediated suppression of TDP43 in primary motor neurons resulted in significantly increased mitochondrial length and density in dendrites (Wang et al., 2013). In addition, co-expression of MFN2 with mutant TDP43 completely prevented all TDP43-induced mitochondrial abnormalities (Wang et al., 2013).

Informative findings have also arisen from work in *Drosophila*. Khalil et al. (2017) found that overexpression of human wild-type TDP43 in neurons resulted in abnormally small mitochondria. The mitochondrial fragmentation was correlated with a specific decrease in the levels of Marf, the MFN ortholog in *Drosophila*. Importantly, overexpression of Marf or inactivation of pro-fission Drp1 ameliorated the defects (Khalil et al., 2017). Similar mitochondrial dysfunctions were observed in another *Drosophila* study, and likewise the mitochondrial fission defects were rescued by co-expression of mitochondrial pro-fusion genes Marf, Opa1, and the dominant negative mutant form of Drp1 (Altanbyek et al., 2016).

Using immunoprecipitation from cortical human brain tissue, TDP43 was found to also interact directly with pro-fusion factor MFN2 (Davis et al., 2018). Knocking down TDP43 in HEK293T cells led to a reduction in MFN2 expression levels, whereas TDP43 overexpression marginally increased MFN2 levels (Davis et al., 2018). Previously, MFN2 repression was shown to inhibit mitophagy and result in the accumulation of damaged mitochondria in muscles during aging (Sebastian et al., 2016), indicating that changes in the balance of mitochondrial fission/fusion machinery affect not only architecture dynamics but mitophagy as well.

Under steady-state conditions, PTEN-induced kinase 1 (PINK1), a mitochondrial serine/threonine kinase, is imported in the inner mitochondrial membrane where it is cleaved by the serine protease presenilin-associated rhomboid-like (PARL) (Yamano and Youle, 2013). Following cleavage, PINK1 is released into the cytosol where it is recognized by the N-end rule E3 enzymes, ubiquitin protein ligase E3 component N-Recognin 1 (UBR1), UBR2, and UBR4 for constitutive and rapid proteasome-mediated degradation (Yamano and Youle, 2013). When mitochondria are damaged, PINK1 is not cleaved and is subsequently anchored to the outer mitochondrial membrane where it recruits and activates, via phosphorylation, the E3 ubiquitin ligase PRKN to trigger selective mitophagy (Pickrell and Youle, 2015). Both PINK1 and PRKN exhibit mutations that have been linked to autosomal recessive early-onset Parkinson’s disease (PD) (Kitada et al., 1998; Hatano et al., 2004; Rohe et al., 2004; Valente et al., 2004).

Using human TDP43 knock-in flies, TDP43-infected mouse primary neurons, TDP43-transfected HEK293T cells, and TDP43^*Q*331*K*^ transgenic mice, Sun et al. (2018), showed that TDP43 downregulates *PRKN* mRNA and protein levels via mechanisms requiring both the RNA-binding and the protein-protein interaction functions of TDP43. Unlike *PRKN*, TDP43 did not regulate *PINK1* at the mRNA level. Instead, overexpression of TDP43 lead to cytosolic aggregates of cleaved PINK1 due to impaired proteasomal activity, and compromised mitochondrial respiration (Sun et al., 2018). Upregulation of PRKN expression or RNAi-mediated downregulation of PINK1 levels suppressed TDP43-induced degenerative phenotype in *Drosophila*, indicating that PRKN and PINK1 are important components of TDP43-induced proteinopathy (Sun et al., 2018). Additionally, it has been reported that accumulation of cleaved PINK1 induces non-selective mitophagy and non-apoptotic cell death (Lim et al., 2015). In this article, it is shown that cleaved PINK1 cytosolic aggregates trigger PRKN translocation to healthy mitochondria, leading to non-selective mitophagy (Lim et al., 2015).

In another study, PRKN was shown to ubiquitinate nuclear TDP43, and together with HDAC6, promote cytosolic TDP43 accumulation reminiscent of ubiquitinated wild-type or mutant TDP43 found in the cytosol in several neurodegenerative diseases (Hebron et al., 2013). Moreover, *Prkn* knockout mice exhibited high levels of TDP43, underscoring an indispensable role for PRKN in mediating TDP43 clearance and cytosolic localization (Wenqiang et al., 2014).

A dual regulation of mitophagy and apoptosis by PRKN via VDAC1, a direct partner of TDP43 in mitochondria (Davis et al., 2018), has also been revealed. Previously, VDACs have been shown to mediate mitophagy via recruitment of PRKN in the mitochondria (Geisler et al., 2010; Sun et al., 2012; Li et al., 2014). More recently, PRKN was shown to mono- or poly-ubiquitinate VDAC1. Polyubiquitination was required for PRKN-mediated mitophagy, whereas mono-ubiquitination was required for mitochondrial calcium influx and apoptosis (Ham et al., 2020). The role of TDP43 in the mono- or poly-ubiquitination of VDAC1 by PRKN has yet not determined.

## FUS

Mutations in the FUS or translocated in liposarcoma (*FUS/TLS*) gene give rise to familial ALS and occasionally FTLD-FUS, both displaying FUS-positive inclusions (Ravanidis et al., 2018). Interestingly, however, in the majority of FTLD-FUS cases, no *FUS* mutations have been identified, but rather an increase in wild-type FUS expression highlighting a dose-dependent role in neurodegeneration (Sabatelli et al., 2013; Deng et al., 2015). Several systems have been used to model FUS-proteinopathies, in all of which wild-type or ALS-mutant FUS overexpression led to progressive neurodegeneration reiterating findings in patients (Huang et al., 2011; Ravanidis et al., 2018).

**FIGURE 2:**
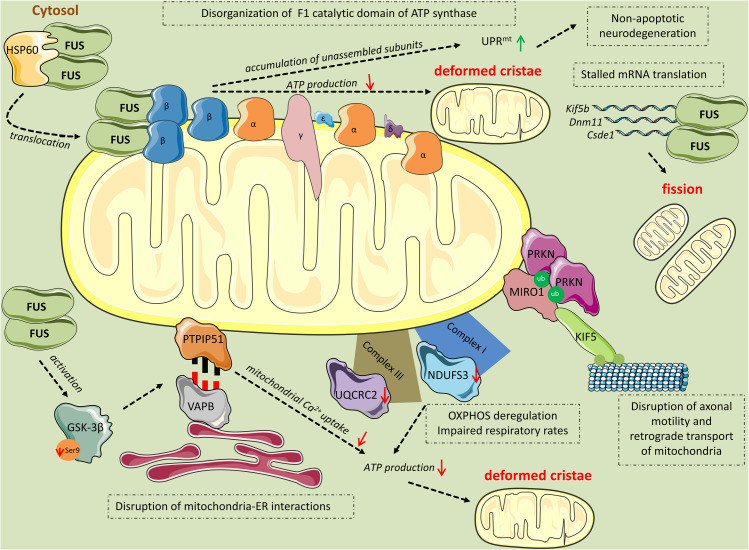
Mitochondrial perturbations induced by FUS. HSP60 mediates FUS translocation to the outer mitochondrial membrane. Mitochondrial-localized FUS binds to the β subunit of the F1 catalytic domain of ATP synthase (Complex V). The binding leads to disassembly of the F1 domain and accumulation of unassembled ATP synthase subunits, including ATP5B, which activates the UPR^*mt*^ response leading to non-apoptotic cell death. Additionally, disruption of the F1 domain of the ATP synthase complex results in impaired ATP production and thereafter, deformed cristae. FUS induces mitochondrial perturbations in several other manners while being in excess in the cytoplasm. Mutant FUS binds to mature mRNAs coding for important mitochondrial proteins including *Kif5b*, *Dnm1l*, and *Csde1*, inhibiting their translation. This inhibition progressively leads to mitochondrial fission. Excess FUS drives the accumulation of PINK1 and PRKN proteins. As a consequence, RHOT1, also known as Miro1, a component of the primary motor/adaptor complex that anchors kinesin to the mitochondrial surface and a direct target of PRKN, is ubiquitinated leading to disruption in axonal motility and retrograde transport of mitochondria. Additionally, FUS has an impact on the OXPHOS process by deregulating the expression of the subunits NDUFS3 and UQCRC2 of Complexes I and III, respectively. OXPHOS deregulation leads to respiratory impairment and subsequent ATP production deterioration and deformed cristae. Finally, FUS decreases the levels of ser9 phosphorylation in GSK-3β, leading to increased GSK-3β activity. Activated GSK-3β deregulates the interaction of mitochondrial tethered membrane protein PTPIP51 and the inner protein of the ER, VAPB, disrupting mitochondria-ER associations. The ER-mitochondria disruption decreased Ca^2+^ uptake by mitochondria following release from ER stores, resulting in reduced ATP production and deformed mitochondria.

Several studies implicate mitochondrial damage as an early event that precedes cell death in FUS proteinopathies (Deng et al., 2015, 2018; So et al., 2018; Figure 2). Deng et al. (2015) showed that overexpression of wild-type or ALS-associated mutant FUS in HEK293 cells reduced the mitochondrial membrane potential and increased the production of mitochondrial ROS. Increased levels of ROS drive mitochondrial translocation of the pro-fission protein DRP1 in ASTCa1 cells, leading to mitochondrial fragmentation (Wu et al., 2011). Likewise, Deng et al. (2015) observed mitochondrial fragmentation in wild-type or mutant FUS-overexpressing HT22 cells, cultured neurons, and transgenic fly motor neurons. They then performed EM to compare healthy control and FTLD-FUS brain mitochondria. While in controls most mitochondria appeared healthy with well-organized cristae as packed-stacks of membrane sheets and with only a few FUS-immunostaining signals, in FTLD patients mitochondria displayed a marked loss or disruption of cristae with frequent detection of “onion-like” deformed shapes and FUS-immuno-positive signals, in close association with the mitochondria (Deng et al., 2015).

Similarly, So et al. (2018), using transgenic hFUS mice, revealed that FUS, which is abundant at the pre-synaptic terminal of the neuromuscular junction (NMJ), caused a significant decrease in the number of mitochondria, while many of those that remained had pronounced abnormalities including disorganized cristae and large vacuoles as early as postnatal day 15. Interestingly, mitochondria in the post-synaptic muscle endplate were abundant and of normal appearance, consistent with other studies demonstrating that mitochondria at distal axon terminals undergo the earliest damage in the course of ALS disease (Magrane et al., 2012; Ruffoli et al., 2015).

Deng et al. (2015) moved on to demonstrate that heat shock protein 60 kDa (HSP60), an ATP-dependent mitochondrial chaperone, interacted with FUS and mediated FUS mitochondrial localization. siRNA-based downregulation of HSP60 levels reduced mitochondrially localized FUS without altering its overall cellular levels; in fact, levels of nuclear and cytoplasmic FUS increased as a result. Accordingly, HSP60 downregulation increased the size of mitochondria and partially rescued mitochondrial defects as well as neurodegenerative phenotypes caused by wild-type or mutant FUS overexpression in transgenic fly photoreceptors. Finally, they found that HSP60 protein levels were elevated in the brains of FTLD-FUS patients (Deng et al., 2015). These observations indicate that HSP60 plays an important role in mediating the translocation of excess FUS in mitochondria, a critical early step in mitochondrial impairment and thereafter neurodegeneration.

Additional mechanisms by which FUS induces mitochondrial damage have been brought forward. Wild-type or mutant FUS were found to interact with the mitochondrial ATP synthase β-subunit (ATP5B) (Deng et al., 2018), which is the essential catalytic subunit of mitochondrial ATP synthase (Wang and Oster, 1998). FUS binding to ATP5B disrupted the assembly of ATP synthase super-complex, suppressing ATP synthesis (Deng et al., 2018). Previously, ATP synthase complex assembly has been closely associated with mitochondrial cristae formation (Paumard et al., 2002). ATP synthase mutants show disorganized cristae in yeast (Paumard et al., 2002; Strauss et al., 2008), which could explain the disruption or loss of cristae observed following FUS overexpression (Deng et al., 2015, 2018; So et al., 2018).

On top of that, whereas ATP synthase complex activities and formation were decreased, mitochondrial ATP5B protein levels were increased in FUS-overexpressing HEK293 cells and flies (Deng et al., 2018). This has given rise to an accumulation of unassembled ATP synthase subunits, including ATP5B, which activated the mitochondrial unfolded protein response (UPR^*mt*^) (Deng et al., 2018). UPR^*mt*^ is an adaptive mechanism to ensure mitochondrial proteostasis and quality control. However, excessive activation of UPR^*mt*^ following severe or extended mitochondrial stresses can induce non-apoptotic neurodegeneration (Martinez et al., 2017). That is likely the case here, as downregulation of UPR^*mt*^ genes ameliorated wild-type or mutant FUS-induced retinal degeneration in flies (Deng et al., 2018).

A different perspective was brought forward by Stoica et al. (2016). They found that wild-type or ALS-associated mutant FUS decreased the endoplasmic reticulum (ER)-mitochondria associations in NSC34 motor neuron cells and in spinal cord motor neurons from FUS transgenic mice (Stoica et al., 2016). Specifically, they showed that FUS disrupted the interaction between the integral ER protein, vesicle-associated membrane protein-associated protein B (VAPB), and the outer mitochondrial membrane protein, protein tyrosine phosphatase interacting protein 51 (PTPIP51) that serve as scaffolds to tether the two organelles (De Vos et al., 2012). This disruption was accompanied by a decrease in Ca^2+^ uptake by the mitochondria following its release from ER stores. Since mitochondrial ATP production is linked to Ca^2+^ levels (De Vos et al., 2012), uncoupling of ER-mitochondria by FUS resulted in impaired ATP production (Stoica et al., 2016). Immunoprecipitation revealed that FUS did not bind either VAPB or PTPIP51. Instead, FUS reduced the inhibitory phosphorylation of ser9 in GSK-3β, resulting in its activation (Stoica et al., 2016). Previously, the same group has shown that GSK-3β inhibition increases the VAPBPTPIP51 interaction; however, the precise mechanism is not yet determined (Stoica et al., 2014). Hence, using the GSK-3β inhibitors AR-A014418 and CT99021H, they showed that FUS-induced defects in ER-mitochondria association as well as mitochondrial Ca^2+^ levels were restored (Stoica et al., 2016). Considering that damaged ER-mitochondria associations have also been described in AD and PD (Zampese et al., 2011; Cali et al., 2012; Hedskog et al., 2013; Ottolini et al., 2013; Guardia-Laguarta et al., 2014), this indicates that perturbation of the ER–mitochondrial axis may be a general feature in neurodegeneration.

Another way by which disease-causing FUS mutations induce mitochondrial impairment and neurotoxicity was deciphered by Nakaya and Maragkakis (2018). They showed that unlike wild-type FUS that predominantly binds pre-mRNAs, the ALS-associated R495X FUS mutant binds mature mRNAs in the cytoplasm (Nakaya and Maragkakis, 2018). Although R495X binding had only a moderate effect on mRNA levels, it significantly reduced the translation of mRNAs that are associated with mitochondrial function such as *Kif5b*, *Dnm1l*, and *Csde1* (Nakaya and Maragkakis, 2018). These alterations were accompanied by a reduction in mitochondrial size, as previously reported (Deng et al., 2015, 2018; So et al., 2018). Importantly, by introducing multiple mutations in the RRM RNA-binding domain of R495X FUS, to reduce its RNA-binding ability (Daigle et al., 2013), they partially abrogated R495X-induced effects on mRNA translation, mitochondrial size, and neurotoxicity, uncovering a novel RNA-mediated pathway of FUS proteinopathy (Nakaya and Maragkakis, 2018).

Insights into the role of PRKN in FUS-mediated mitochondrial dysfunction were revealed by Cha et al. (2020). Using *Drosophila* flies, they showed that when PRKN was co-overexpressed with FUS, it was able to rescue locomotive defects (Cha et al., 2020). At the cellular level, PRKN co-overexpression did not lead to any significant mitochondrial morphological improvements compared to the flies only overexpressing FUS; in fact, PRKN overexpressed alone also exhibited fragmented mitochondria (Cha et al., 2020). Instead, they found that PRKN restored the expression of mitochondrial subunits I (NDUFS3) and III (UQCRC2), which are significantly decreased in FUS-induced ALS flies. As a result, flies overexpressing both FUS and PRKN had partially restored ATP levels (Cha et al., 2020). Interestingly, complex III is one of the five mitochondrial distinct multi-subunit complexes (I–V) whose activity is reported to be dampened in spinal cord tissues of ALS patients (Sasaki and Iwata, 2007). Taken together, these observations demonstrated a protective role of PRKN in FUS-induced mitochondrial dysfunction.

Contradictory findings concerning the role of PRKN in FUS-mediated defects have also been reported (Chen et al., 2016). Overexpression of wild-type or mutant FUS in HEK293 cells lead to the accumulation of PINK1 and PRKN proteins (Chen et al., 2016). As a consequence, the Ras homolog family member T1 (RHOT1, also known as Miro1), a component of the primary motor/adaptor complex that anchors kinesin to the mitochondrial surface and a direct target of PRKN, was ubiquitinated leading to the disruption in axonal motility and retrograde transport of mitochondria (Chen et al., 2016). Previously, Miro1 was shown to be phosphorylated by PINK1, which promoted its proteasomal degradation by PRKN (Wang et al., 2011; Liu et al., 2012). RNAi-mediated downregulation of both PINK1 and PRKN restored locomotive defects in FUS transgenic flies (Chen et al., 2016). As the PINK1/PRKN pathway also promotes mitochondrial fission (Poole et al., 2008; Yu et al., 2011), Chen et al. (2016) proposed that the upregulation of PINK1 and PRKN is partly responsible for mitochondrial fragmentation induced by wild-type and mutant FUS overexpression.

## TIA1 and TIAR

T-cell intracellular antigen 1 and TIA-related/like protein share an extended identity in the amino acid sequence, and like other RBPs, they translocate to the cytoplasm following cellular stress conditions forming stress granules (SG) (Ravanidis et al., 2018). Missense mutations in the *TIA1* gene cause both Welander distal myopathy (WDM) (Hackman et al., 2013) and ALS, characterized by delayed SG disassembly and accumulation of non-dynamic SGs that harbor TDP43 (Mackenzie et al., 2017).

**FIGURE 3:**
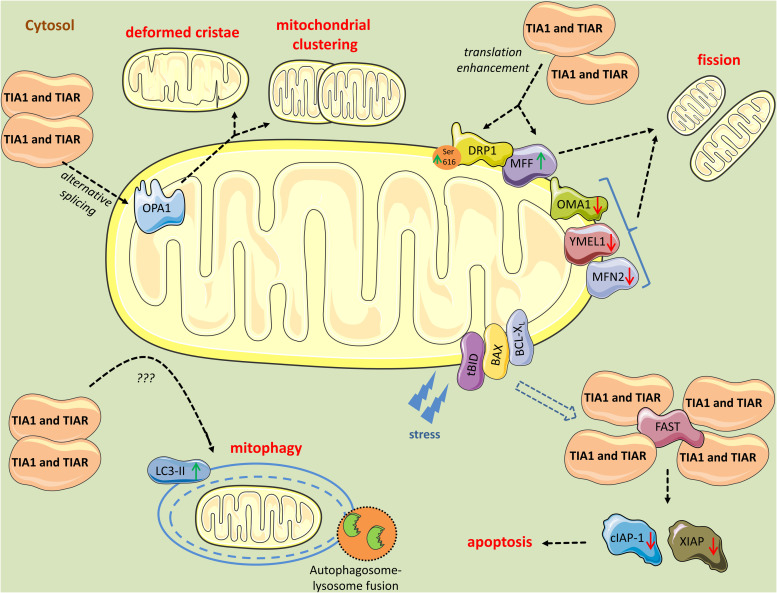
Mitochondrial perturbations induced by TIA1 and TIAR. TIA1 and TIAR mediate exon 4b inclusion in the pre-mRNA of *OPA1* generating the OPA1 variant 5, which is associated with a smaller mitochondria, mitochondrial clustering, and remodeling around the perinuclear region. Further, cytosolic TIA1 enhances the translation of *MFF* mRNA and promotes DRP1 translocation to mitochondria leading to mitochondrial fragmentation. In parallel, TIA1 and TIAR induce modest downregulation of the pro-fusion proteases OMA1, YMEL1, and MFN2, further contributing to the pro-fission phenotype and mitophagy. TIA1 also has pro-apoptotic properties inhibited by FAST. FAST is released from its mitochondrial tether during stress, a process mediated by tBID and BAX. Following its release, FAST binds to TIA1 and prevents TIA1-mediated silencing of mRNAs encoding inhibitors of apoptosis, such *cIAP-1* and *XIAP*. When TIA1 is in excess, it binds FAST and obstruct its anti-apoptotic events. Finally, TIA1 and TIAR increase LC3-II levels, yet the mechanism is unknown, leading to increased mitophagic events.

Early in the analysis of TIA1 cell models, it became evident that TIA1 and TIAR affect mitochondrial dynamics (Figure 3). Using EM, Carrascoso et al. (2017) found that TIA1 or TIAR overexpression in HEK293 cells promoted mitochondrial clustering and fission. Closer inspection of mitochondria revealed changes in cristae organization, with many cristae having a slightly wider and more loosely organized intermembrane space than those of control cells (Carrascoso et al., 2017). Further, the mtDNA/nDNA ratio was similar between control and TIA1- or TIAR- overexpressing cells, suggesting that the changes in mitochondria were linked to reorganization dynamics rather than *de novo* mitochondrial biogenesis. Mitochondrial respiration and ATP production were impaired as a result (Carrascoso et al., 2017). When switched from glucose to galactose or fatty acids as cell culture substrates, to promote a switch from glycolysis to OXPHOS and determine the degree of mitochondrial dependency in cell growth, TIA1- or TIAR- overexpressing cells showed reduced proliferation rates (Carrascoso et al., 2017). Additionally, they displayed increased mitophagy rates and ROS production. Enhanced cleaved poly (ADP-ribose) polymerase 1 (PARP1) levels and delay in G1/S cycle phase transition, phenomena of early apoptosis, correlated with increased mitophagy (Carrascoso et al., 2017). Increased mitochondrial DNA damage were also observed in TIA1- or TIAR- overexpressing cells following H_2_O_2_ treatment suggestive of impaired antioxidant defense (Carrascoso et al., 2017). Collectively, these results indicate that TIA1 or TIAR provoke respiratory deficiency and compromised mitochondrial function (Carrascoso et al., 2017).

Mechanistically, TIA1 and TIAR mediated exon 4b inclusion in the pre-mRNA of *OPA1* generating the OPA1 variant 5. OPA1 is a dynamin-like GTPase that regulates cristae junction numbers and stability, and the different OPA1 protein isoforms (eight in humans) relay instructions that help determine fusion, build cristae, and tune the morphology of mitochondria (Olichon et al., 2007; Song et al., 2007; Glytsou et al., 2016). OPA1v5, specifically, promotes mitochondrial clustering and remodeling around the perinuclear region (Song et al., 2007; Carrascoso et al., 2017). Ablation of TIA1 or TIAR in mouse embryonic fibroblasts (MEFs) favored the expression of short forms of OPA1, and the appearance of elongated mitochondria indicative of fusion phenotypes (Carrascoso et al., 2017). Furthermore, knockdown of OPA1 or overexpression of OPA1v5 triggered mitochondrial clustering mimicking TIA1 or TIAR effects (Carrascoso et al., 2017). In addition, proteases associated with fusion (OMA1, YMEL1, and MFN2) were modestly downregulated in TIA1- or TIAR-overexpressing cells, whereas the fission-associated protein MFF was slightly upregulated, further contributing to the pro-fission phenotype (Carrascoso et al., 2017).

Tak et al. (2017) independently reported similar mitochondrial phenotypes following TIA1 modulation, but provided different mechanistic insights. Likewise, they showed that TIA1 overexpression in CHANG liver cells enhanced mitochondrial fission, while downregulation enhanced mitochondrial elongation. In addition, TIA1 downregulation increased mitochondrial activity, including the rate of ATP synthesis and oxygen consumption (Tak et al., 2017). Further, they identified *MFF* 3’UTR as a direct target of TIA1 and showed that TIA1 promoted mitochondrial fragmentation by enhancing MFF translation. Accordingly, *Tia1*-null MEF cells had decreased levels of MFF and mitochondrial DRP1, thereby leading to mitochondrial elongation (Tak et al., 2017).

Studies investigating the p.E384K mutant form of TIA1 (TIA1^*WDM*^) responsible for WDM revealed similar findings (Carrascoso et al., 2019). TIA1^*WDM*^ overexpression in HEK293 cells resulted in mitochondrial fission and mitochondrial swelling with an abnormal distribution of cristae. This led to decreased mitochondrial membrane potential and enhanced redox status (Carrascoso et al., 2019). Additionally, there was an increase in the formation of autophagosomes and autolysosomes, as well as mitophagic and apoptotic rates (Carrascoso et al., 2019). Taken together, these results revealed that similar to wild-type TIA1, disease-associated mutant TIA1 overexpression has a negative impact on mitochondrial dynamics, leading to mitochondrial dysfunction and cell death.

Sanchez-Jimenez and Izquierdo (2013) used *Tia1* and *Tiar* knock-out MEFs to study the molecular and cellular consequences. They found that TIA1 and TIAR knockout cells had two to threefold more mitochondria, six to sevenfold higher mitochondrial membrane potential, and twofold higher ROS levels. Mitochondria had atypical morphology, with some being enlarged and others being fragmented (Sanchez-Jimenez and Izquierdo, 2013). These alterations were associated with nuclear DNA damage, revealed by 8-hydroxy-2′-deoxyguanosine (8-oxo-dG) staining, and high rates of autophagy, possibly as a compensatory mechanism toward survival. Consequently, TIA1 and TIAR knockout MEFs displayed defects in cell proliferation and increased cell death (Sanchez-Jimenez and Izquierdo, 2013).

A different perspective by which TIA1 is promoting apoptosis was brought forward by Li et al. (2004b). They proposed that during stress, TIA1 silences (Kedersha et al., 2000; Anderson and Kedersha, 2002), among others, the translation of mRNAs encoding inhibitors of apoptosis, and that the Fas-activated serine/threonine kinase (FAST) phosphoprotein is counteracting this function (Li et al., 2004b). They showed that FAST, which is tethered to the outer mitochondrial membrane in association with BCL-X_*L*_ (Li et al., 2004a), is a constitutive pro-survival protein (Li et al., 2004b). RNAi-mediated silencing of endogenous FAST in HeLa cells resulted in apoptosis, whereas overexpression of FAST inhibited both Fas- and UV- induced apoptosis (Li et al., 2004b). Mechanistically, they found that a FAST mutant lacking its TIA1-binding domain did not inhibit apoptosis, and overexpressed TIA1 inhibited the antiapoptotic effects of FAST. They proposed that in response to stress, tBID and BAX move to the outer mitochondrial membrane, where they sequester BCL-X_*L*_, releasing FAST from its mitochondrial tether. FAST then binds to TIA1 and prevents TIA1-mediated silencing of mRNAs, including those encoding inhibitors of apoptosis, such as *cIAP-1* and *XIAP* (Li et al., 2004b). Hence, FAST serves as a cellular sensor of mitochondrial stress, that in response to stress, modulates TIA1-regulated posttranscriptional silencing responses.

## Pumilio

Pumilio belongs to the evolutionary conserved Pumilio and FBF (PUF) family of RBPs comprised two paralogous members in vertebrates (Pum1 and 2), and one in *Drosophila* (Pum). It is an important mediator of neurological processes, including olfactory learning and motor function (Ravanidis et al., 2018). In humans, a *PUM1* mutation is associated with adult-onset ataxia, whereas haploinsufficiency due to deletions or missense variants cause developmental delay and seizures (Gennarino et al., 2018).

**FIGURE 4:**
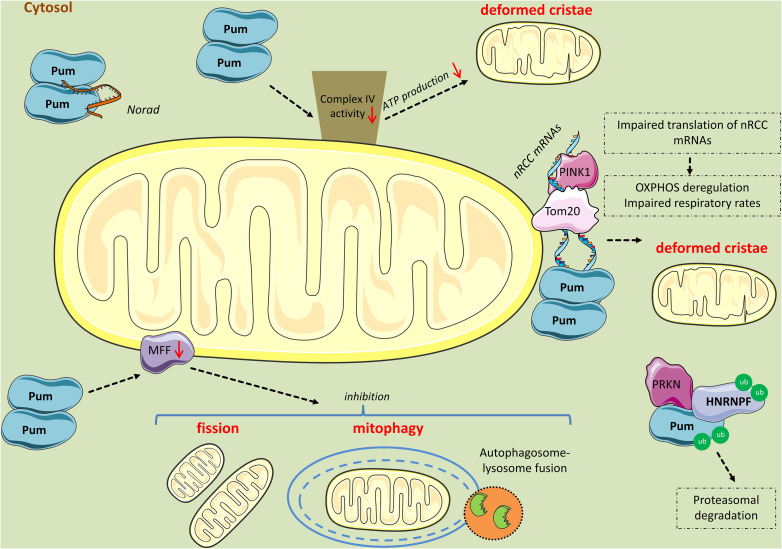
Mitochondrial perturbations induced by PUM2. PUM2 reduces cytochrome c oxidase complex (Complex IV) activity, leading to impaired respiration and deformed cristae. Interestingly, the long non-coding RNA *NORAD* inhibits PUM2 function by sequestering PUM2 from binding to mitochondrial mRNA targets. Further, PINK1 in association with Tom20 promote the expression of nuclear-encoding mitochondrial (*nRCC*) mRNAs in the outer mitochondrial membrane by competing with PUM and other translation repressors. PINK1 competes with PUM for mRNA-binding, while PRKN mono-ubiquitinates PUM and HNRNPF lowering their affinity for *nRCC* mRNAs and possibly leading to their proteasomal degradation. However, when PUM is in excess, it binds to the *nRCC* mRNAs and represses their translation. Finally, PUM2 binds to *MFF* mRNA and represses its translation, leading to reduced fission and mitophagy.

Several study systems ranging from yeast to mice highlighted the role of PUFs in regulating mitochondrial biogenesis and mitophagy (Figure 4). In yeast, Puf3p was shown to specifically associate with 135 mRNAs, 87% of which are nucleus-encoded mitochondrial mRNAs (Gerber et al., 2004). Among these mitochondrial mRNAs, 59% (80 genes) are involved in protein biosynthesis, including structural components of the ribosome; 16% (22 genes) in mitochondrial organization and biogenesis; 13% (17 genes) in aerobic respiration; 9% (12 genes) in mitochondrial translocation; 9% are tRNA ligases (12 genes); and 7% are translational regulators (nine genes) (Gerber et al., 2004). Interestingly, when Puf3p was deleted in yeast, Puf3p-associated mRNAs were not only selectively increased compared to all other mRNAs (Gerber et al., 2004), but also mislocalized away from mitochondria (Eliyahu et al., 2010), indicating that Puf3p regulates the stability and localization of mRNAs expressing mitochondrial proteins. Consequently, yeast strains overexpressing Puf3p exhibited respiratory dysfunction and abnormal mitochondrial morphology and motility (Gerber et al., 2004; Garcia-Rodriguez et al., 2007).

Studies in *Drosophila* backed yeast findings. Work by Gehrke et al. (2015), revealed that nuclear mRNAs encoding respiratory chain complexes (*nRCC*) are localized in a PINK1/Tom20-dependent manner to the mitochondrial outer membrane, where they are de-repressed and translated by PINK1/PRKN pathway through the displacement of translation repressors, including PUM and hnRNPF; in this case, PINK1 displayed an RNA-binding capacity competing with PUM for mRNA-binding, while PRKN mono-ubiquitinated the RBPs lowering their affinity for *nRCC* mRNAs (Gehrke et al., 2015). Accordingly, inhibiting PUM via RNAi was found to increase, whereas PUM overexpression decreased *nRCC* mRNAs abundance (Gehrke et al., 2015). In addition, PUM inhibition rescued ATP production, mitochondrial morphology, and neuromuscular-degeneration phenotypes of PINK1, but not PRKN mutant flies (Gehrke et al., 2015). Collectively, these findings revealed that besides its well-known serine/threonine kinase activity, PINK1 is also an RBP competing with PUM to control mitochondria biogenesis (Gehrke et al., 2015).

Electron microscopy of skeletal muscles from PUM2-overexpressing mice revealed the accumulation of subsarcolemmal, irregularly shaped and abnormally enlarged mitochondria lacking normal cristae (Kopp et al., 2019). Furthermore, a global reduction in cytochrome c oxidase (complex IV, COX) activity was observed. In addition, transient expression of PUM2 in MEFs or stable expression of either PUM1 or PUM2 in HCT116 cells significantly impaired respiration, providing compelling evidence that PUM hyperactivity results in mitochondrial dysfunction (Kopp et al., 2019). Interestingly, a non-coding RNA called NORAD acts as a guardian of the transcriptome by being a preferred target of PUM2, thereby inhibiting its translation suppressive functions (Kopp et al., 2019).

Research findings from D’Amico et al. (2019) associated PUM2 with aging defects via impaired mitochondrial dynamics. They reported that elevated levels of PUM2 are found in muscle and neocortex of aged mice (Edwards et al., 2007; Oberdoerffer et al., 2008; D’Amico et al., 2019) as well as muscle biopsies of aged humans (Yang et al., 2015). Additionally, *Pum2* levels in the brains of mice strains BXD and LXS are inversely correlated with longevity (Gelman et al., 1988; Liao et al., 2010). To experimentally validate this suggestive effect on lifespan, they used *Caenorhabditis elegans* to show that PUF8, ortholog of PUM2, knockdown was associated with increased lifespan (D’Amico et al., 2019). Consistently, knock-down of *puf8* and *Pum2* improved both basal and maximal respiration in old worms and mouse myoblasts, respectively (D’Amico et al., 2019). Like in previous studies, using multi-tissue gene set enrichment analysis (GSEA) in the human GTEx cohort, they found that PUM2 expression levels were inversely correlated with clusters of genes responsible for mitochondrial function, including genes important for OXPHOS and cellular respiration (D’Amico et al., 2019). Furthermore, from CLIP-Seq data (Hafner et al., 2010), they identified a perfect PUM2 site in the 3′UTR of *MFF* mRNA and showed that PUM2 negatively regulated *MFF* translation in C2C12 and HeLa cells (D’Amico et al., 2019). Consequently, *Pum2* silencing increased the percentage of C2C12 cells undergoing fission and mitophagy, and this was reversed by simultaneously performing *Mff* RNAi. Similarly, *puf8* depletion improved mitochondrial homeostasis during nematode aging and canceled by *mff1* co-depletion (D’Amico et al., 2019). Lastly, *Pum2* depletion using CRISPR-Cas9 in the muscle of old mice increased MFF levels and mitophagy, and improved respiration. Collectively, these data suggest that PUM2 regulates mitochondrial dynamics and mitophagy via MFF.

## Conclusion

Over the years, several lines of evidence have implicated mitochondrial dysfunctions in many diseases, particularly those associated with neurodegenerative disorders and aging. Following recent findings that mutations or misexpression of RBPs can cause neurological impairments, there has been tremendous interest in identifying their molecular pathogenetic mechanisms. Interestingly, it turned out that mitochondria are also direct and early targets of RBP misregulation reiterating their importance for cellular homeostasis. These findings suggest that pharmaceutical agents improving mitochondrial life cycle can be attractive therapeutics for easing mitochondrial dysfunction in these diseases.

## Author Contributions

SR and ED wrote and edited the manuscript.

## Conflict of Interest

The authors declare that the research was conducted in the absence of any commercial or financial relationships that could be construed as a potential conflict of interest.
